# Twelve-Month Studies on *Perilla* Oil Intake in Japanese Adults—Possible Supplement for Mental Health

**DOI:** 10.3390/foods9040530

**Published:** 2020-04-22

**Authors:** Michio Hashimoto, Kentaro Matsuzaki, Setsushi Kato, Shahdat Hossain, Miho Ohno, Osamu Shido

**Affiliations:** 1 Department of Environmental Physiology, Shimane University Faculty of Medicine, Izumo 693-8501, Japan; matuzaki@med.shimane-u.ac.jp (K.M.); shahdat@juniv.edu (S.H.); o-shido@med.shimane-u.ac.jp (O.S.); 2 Kato Hospital, Jinjukai Healthcare Corporation, Kawamoto, Shimane 696-0001, Japan; katosetsu@k-jinju.or.jp (S.K.); ohno@k-jinju.or.jp (M.O.); 3 Department of Biochemistry and Molecular Biology, Jahangirnagar University, Savar, Dhaka 1342, Bangladesh

**Keywords:** *perilla* oil, α-linolenic acid, mental health, depression, apathy, ω-polyunsaturated fatty acid

## Abstract

*Perilla* oil (PO), rich in α-linolenic acid (LNA, C18:3, ω-3), is increasingly alleged to have numerous health benefits in humans. However, the current reports detailing the effects of PO on human mental health are not adequate. Therefore, in the current investigation we compared the effects of PO or placebo treatment on the mental condition of healthy adult Japanese volunteers. At baseline and after 12 months of treatment, mental health condition was assessed using the Zung Self-Rating Depression Scale (SDS) and Apathy Scale, and serum biochemical parameters were determined. From baseline to 12 months of intervention, both SDS depression and apathy scores improved significantly in the PO-administered group. Compared to those of control group, serum norepinephrine and serotonin levels after 12 months decreased in the PO-administered group. The enhanced mental state observed in PO-subjects was accompanied by LNA level increases in erythrocyte plasma membranes. Our data demonstrate that PO intake enhances blood LNA levels and may maintain healthy mental conditions in adult subjects.

## 1. Introduction

Numerous reports suggest that ω-3 polyunsaturated fatty acids (PUFAs) have major roles in the maintenance and sustenance of brain cognition in both animals and humans [1,2,3]. Fats and oils, such as docosahexaenoic acid (DHA), can enrich the brain with essential fatty acids, and dietary lipids therefore appear to be an important factor for brain fatty acid nutrition. Many studies support the notion that dietary lipid selection significantly correlates with human ailments such as obesity, atherosclerosis, hypertension, stroke, diabetes, and brain cognition [4]. Furthermore, high fat diet increases brain neuroinflammation [5], which can also affect cognition. The increase in ω-6/ω-3 PUFA ratios found in western diets is associated with the increased incidence of depression [6]. Tsuboi et al. (2013) [7] reported that increased levels of serum saturated fatty acids and ω-6 PUFAs such as arachidonic acid (AA, C22:4, ω-6), are associated with depression. On the contrary, ω-3 PUFAs are involved in the attenuation of neuro-inflammation [8], prevention of neuronal death [9], and development of cognitive ability [10,11] and improvement of mood disorders in humans and animals [12,13,14]. These reports support the concept that brain inflammation, depression, and anxiety seen in patients suffering from mental disorders may be related to a deficiency in ω-3 PUFAs.

*Perilla* oil (PO) is considered a rich source of ω-3 PUFAs, as it is comprised chiefly of α-linolenic (LNA; 18:3, ω-3; 54–64%) and linoleic (13–20%) acid. PO also contains 12–22% monounsaturated oleic acid (OLA), and a small percentage of saturated fatty acids such as palmitic (PLA; 5–7%) and stearic (STA; 1–3%) acid. Notably, amongst the plant seed-based oils, PO possesses the highest proportion of ω-3 fatty acids, the most notable of which is LNA [15]. Other than acting as an anti-thrombotic [16], antiarrhythmic [17] and anti-inflammatory [18,19] fatty acid, LNA is believed to have neuroprotective effects [20]. LNA intake has previously been associated with reduced incidence of myocardial infarction [21], atherosclerosis [22], and mortality from coronary artery disease [23]. Because of the positive impact of LNA’s metabolites, such as eicosapentaenoic acid (EPA) and DHA, on learning-related memory in young and old rats [10,24] as well as Alzheimer’s disease (AD) in model rats [25,26], linoleic acid (LLA), being the precursor of EPA and DHA, is also gaining increasing interest for improving brain cognition. Direct application of purified LNA to glial cell lines inhibited Amyloid β-induced oxidative stress [27]. Neuronal cell lines pretreated with LNA also inhibited H_2_O_2_-induced apoptotic neuronal cell death [28], thus suggesting that LNA has significant antioxidative potential. However, the reports detailing the effects of PO on human cognitive function and mental health are not adequate. In the current study, we investigated the effects of LNA-rich PO on mental condition in Japanese adults.

## 2. Materials and Methods

### 2.1. Participants

A 12-month randomized, double-blind, placebo-controlled study was conducted among healthy 30–78 years old adults living in Okazaki, Japan. All participants underwent anthropometric, blood biochemistry, and mental health tests. Participants were excluded if they had evidence of a medical disorder including renal, respiratory, cardiac, or hepatic disease, diabetes mellitus, and endocrine, metabolic, or hematological disturbances, or were using any psychotropic drug/supplement that might significantly influence the outcomes of the study, or had a hypersensitivity or allergy to PO.

Participants were divided into two groups: a placebo group (n = 37) who consumed 7.0 mL (contained in an aluminum package) of olive oil daily, and a PO group (n = 38) who consumed 7.0 mL (contained in the identical aluminum package) of PO daily (The composition of PO is shown in Table 1). Neither participants nor researchers knew what capsules were being taken by each individual. Both PO and placebo capsules were identically packaged, and had no difference in appearance, texture, or smell. Both oils were provided by Ota Oil Co. Ltd., (Okazaki, Japan). This study was conducted according to the principles of the Declaration of Helsinki and Good Clinical Practice, and was approved by the Ethics Committee of Kato Hospital (approval number 2015-0017). All participants provided written informed consent before participation. Hospital nurses measured participant body weight, height, and blood pressure. Participants were then asked to answer a self-reported general questionnaire on lifestyle, including questions related to medical history, and a brief diet history questionnaire.

### 2.2. Blood Sampling and Cognitive Evaluations of Mental Health

At baseline, and after 12 months of intervention, blood samples were obtained in the morning or afternoon, after ascertaining that the participants had eaten breakfast or lunch. Serum separated from whole blood samples was stored at −80 °C within 8 h of collection. Additionally, blood was collected following 3 and 6 months of intervention in order to monitor LNA, EPA, and DHA levels in the erythrocyte plasma membranes. Mental health condition was assessed at baseline, 3 months and 12 months using the Japanese version of Starktein’s Apathy Scale and the Zung Self-Rating Depression Scale (SDS) [29,30].

### 2.3. Blood Biochemical Analysis

Serum biochemical variables, glutamate-pyruvate transaminase (GPT), glutamate-oxaloacetate transaminase (GOT), gamma-glutamyl transpeptidase (γ-GT), high-density lipoprotein cholesterol (HDL-C), low-density lipoprotein cholesterol (LDL-C), triglyceride (TG), total cholesterol (TC), albumin (ALB), blood urea nitrogen (BUN), and creatinine (CRE) were measured with an automated analyzer 7700DDPP (Hitachi, Tokyo, Japan). Serum glycated hemoglobin (HbA1c) levels were determined using high-performance liquid chromatography (HPLC; HLC-723G9; TOSOH, Tokyo, Japan), and blood sugar (BS) was measured with an automatic analyzer GA08II (A&T Co., Kanagawa, Japan). Fatty acid composition was determined using gas chromatography, as previously described [31].

Monoamine concentrations, norepinephrine (NE), epinephrine (Epi), dopamine (DA), and 5-hydroxytryptamine (5-HT (serotonin)) in the serum were measured using a previously described HPLC method [32]. Briefly, serum was pretreated with clean column EG (EICOM, Kyoto, Japan). The HPLC equipment consisted of an EICOM HTEC-500 (EICOM), equipped with a data processor (EICOM EPC-500 PowerChrom) and an automatic injector (EICOM M-514). Chromatographic separation was performed using an EICOMPAK CA-5ODS column (2.1 mm × 150 mm ID) linked to a precolumn (EICOM PREPAK PC-03-CA, 3.0 mm ID). PowerChrom software was used for data collection and analysis (EICOM). The mobile phase consisted of 0.1 M phosphate buffer (pH 5.7) containing 12% methanol, 700 mg/L sodium 1-octanesulfonate, and 50 mg/L EDTA disodium salt. The flow rate was 0.23 mL/min, and the applied potential was + 450 mV over an Ag/AgCl reference electrode. Column temperature was maintained at 25 °C.

### 2.4. Statistical Analysis

**Figure 1:**
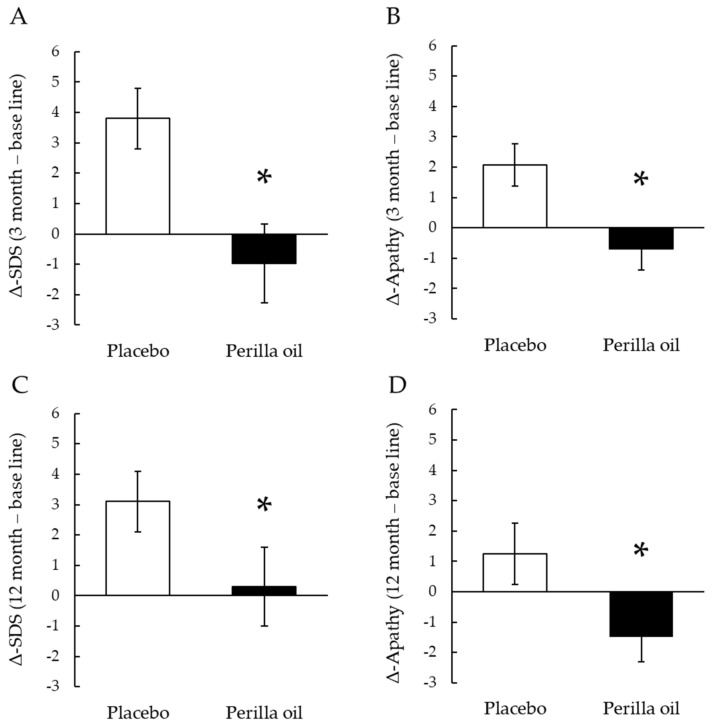
Effect on PO-administration on SDS and Apathy scores. Changes (Δ) in the SDS scores or Apathy scores from baseline to 3 months (**A**,**B**) or 12 months (**C**,**D**) in the PO-intake group and placebo groups. Results are means ± SE. * *p* < 0.05.

**Figure 2:**
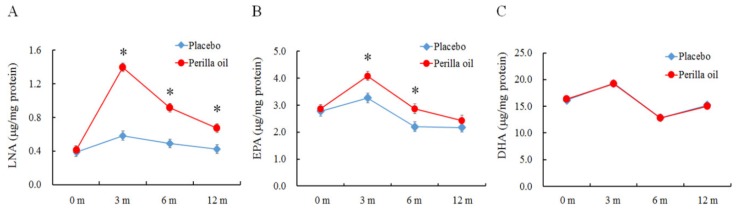
Effect on PO-administration on erythrocyte plasma membrane (**A**) LNA, α-linolenic acid; (**B**) EPA, eicosapentaenoic acid; and (**C**) DHA, docosahexaenoic acid. m: Months. Placebo, n = 34. *Perilla* oil, n = 31. Results are means ± SE. * *p* < 0.05.

Basic fatty acid composition of placebo and *perilla* oil (Table 1) changes (Δ) in the SDS scores or Apathy scores, from baseline to 3 months or 12 months, in the PO-intake group and placebo groups (Figure 1); and fatty acid profile of the erythrocyte plasma membranes at baseline and 12 months (Table 4) were expressed as mean ± SE. Student’s *t*-test was used to evaluate differences in demographic and baseline variables. Participant’s parameters at base line (Table 2) and at 12 months (Table 3) were expressed as mean value, median value with the minimum (Min) and maximum (Max) levels. These values were analyzed by Mann-Whitney U test. Changes in LNA, EPA and DHA levels of erythrocyte plasma membrane every three months were analyzed by Two-way analysis of variance (ANOVA) followed by Bonferroni’s post hoc test (Figure 2). All analyses were carried out using PASW Statistics software (Version 18.0; SPSS Inc.; Chicago, USA). All hypothetical tests were two-sided, and significance was reported as *p* < 0.05.

## 3. Results

### 3.1. Demographic and Clinical Characteristics at Baseline

A total of 75 participants (43 women and 32 men) completed all baseline assessments and were randomized to either the placebo or PO group. A total of 65 participants (86.3%) completed the study in its entirety. Excluding individuals who withdrew from the study did not leave a significant difference in sample size between groups. All baseline characteristics are shown in Table 2. There were no significant between-group differences in sex, age, body weight, belly circumference, body mass index (BMI), body fat, blood pressure, or serum biochemical parameters.

### 3.2. Nutritional Intake

There was no significant difference in mean 12-month dietary nutritional intake between placebo and PO groups. No noticeable side-effects that disturbed daily life (allergic reactions, palpitations, stomach irritation, etc.) were found in either group.

### 3.3. Effect of PO-Administration on Mental Health

The changes (Δ) in SDS and apathy scores from baseline to 3 months were significantly lower in the PO group (Figure 1A, *p* = 0.024; and B, *p* = 0.008). Moreover, Δ-SDS and Δ-Apathy scores in PO group at 12 months were significantly lower than those of placebo group (Figure 1C, *p* = 0.037; 1D, *p* = 0.031). Additionally, a gender difference in Δ-SDS score from baseline to 12 months in the PO and placebo groups was analyzed. Δ-SDS score in women was significantly lower in the PO group than that of placebo group (*p* = 0.041), while no significant difference was observed in men (*p* = 0.459, Supplementary Materials Figure S1).

### 3.4. Effect of PO-Administration on Body Constitution and Serum Biochemical Parameters

There were no significant differences between groups in height, body weight, BMI, or blood pressure at month 12 (Table 3). Correspondingly, the placebo and PO groups did not differ in their blood biochemistry parameters (Table 3).

### 3.5. Effect of PO-Administration on the Fatty Acid Profile of Erythrocyte Plasma Membrane

The erythrocyte plasma membrane fatty acid profile, monitored at baseline and following 12 months of treatment, is shown in Table 4. Other than LNA, which significantly increased over time in PO subjects, erythrocyte plasma membrane fatty acid levels did not change. No fatty acid levels changed over time in placebo-administered subjects.

LNA, EPA and DHA profiles in the erythrocyte plasma membrane measured at baseline and at 3, 6 and 12 months after treatment are shown in Figure 2. Compared to the LNA levels at baseline in the PO group, it significantly increased by 250.0% at 3 months after treatment and decreased after that, but it significantly increased by 130.0% at 6 months and by 67.5% at 12 months (Figure 2A). These levels were significantly higher in the PO group than the placebo group (*F* = 21.823, *p* < 0.0001) at each time point. Similarly, EPA levels significantly increased by 42.7% at 3 months after treatment compared to the EPA levels at baseline and then decreased. At month 12, EPA levels decreased to placebo-comparable levels (Figure 2B). EPA levels at month 3 and 6 were higher in PO subjects than placebo control subjects (*F* = 3.382, *p* < 0.05). The levels of DHA in the erythrocyte plasma membranes remained similar between groups for the entirety of the study (Figure 2C, *p* > 0.05).

### 3.6. Effect of PO-Administration on Serum Monoamine Levels

**Figure 3:**
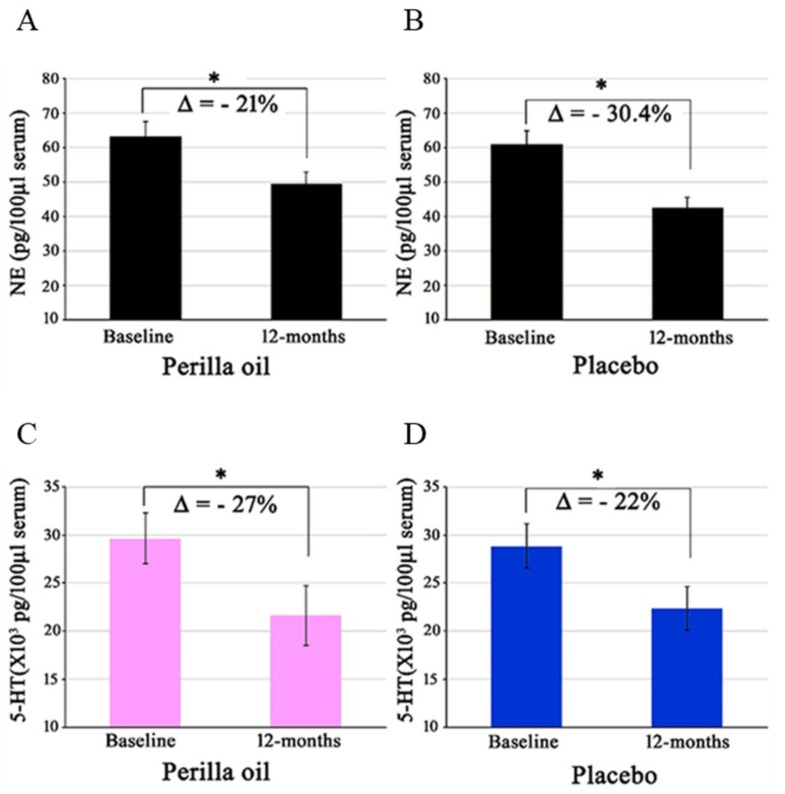
Effect of PO administration on serum norepinephrine (NE) and serotonin (5-HT) levels. There were no inter-group differences, but significant intra-group decreases in serum levels of NE (**A**,**B**) and serotonin (**C**,**D**) were noted in both PO and placebo groups. Δ = Changes from baseline to 12 months. Placebo, n = 34. *Perilla* oil, n = 31, * *p* < 0.05.

From baseline to 12 months of oral PO administration, NE levels decreased by >30% in the placebo group, and 21% in the PO group (Figure 3). The mean change in NE levels from baseline to 12 months was 9% greater in the placebo group. Epi decreased by ~4% in the PO group only. Levels of 5-HT (serotonin) decreased by 27% in the PO group, and 22% in the placebo group (Figure 3). DA levels did not change in either group after intervention.

## 4. Discussion

The results of the present study suggest that PO administration enhances blood LNA and EPA levels which may relieve mental conditions such as depression and apathy, as indicated by decreases in Apathy and SDS scores in PO treated subjects compared to their placebo-treated counterparts. Our 12-month interventional study also concludes that PO administration does not have any negative clinically significant side effects on its users.

The mechanism by which the treatment of PO improved mental condition is not well understood. In general, following digestion and absorption, rises in the levels of dietary components in the erythrocyte membranes reflect their incorporation into cell membranes of other tissues. Consequently, their increased concentrations could be attributed to numerous physiological activities, including brain activity. The levels of LNA in the erythrocyte membranes measured after 3, 6 and 12 months of intervention, as shown in Figure 2, significantly increased. The observed levels of LNA only represent a small percent of total fatty acids in the membranes (Table 4), and its level was highest at month 3. Levels of EPA, an LNA metabolite, were also high following 3 and 6 months of intervention, however, decreased to placebo group-comparable levels after 12 months of treatment. The levels of DHA in the erythrocyte membranes of the PO-intake group did not change during the intervention (Figure 2C). Consistent with our results, consumption of LNA-rich flaxseed oil has previously led to significant increases in tissue EPA, but not DHA [33]. Hamazaki et al. (2006) [34] also reported that PO-intake does not affect the EPA and DHA levels of the human plasma phospholipid fraction. In humans, the antiarrhythmic effect of LNA did not correlate to cell membrane levels of LNA, but correlated rather to its converted metabolites, such as EPA/ DHA, which are usually derived from fish. Dietary LNA does not generally alter DHA levels [35,36,37]. Therefore, the mechanism(s) of LNA currently benefitting the mental condition of our subjects without significantly increasing tissue DHA levels are unknown. We speculate several possibilities. Our PO dose (4 g/day) may be too low, and a higher dose, as used by Finnegan et al. (2003) [38], may have increased tissue LNA levels. Alternatively, enucleated erythrocytes might not be the ideal cell-type to reflect LNA-penetration in other tissue cells. Another thought is that the LNAs contained in the PO may have been exploited for energy sources and/or as substrates for the conversion into lipid mediators through COX/LOX pathways, or via its longer-chained metabolites, such as EPA/DHA. In the present study, serum PO levels in the PO group peaked at month 3 and gradually decreased at month 6 and 12, but the levels at month 12 were significantly higher than that of the control group (Figure 2A). If the amount of PO supplemented is too low and is used as an energy source and/or as substrates of lipid mediators, the intake may need to be increased with a long intake period.

We consider that all these possibilities may coexist. The basis of these speculations are: (i) animals fed LNA-only diets might have brain DHA levels similar to DHA-fed animals [39]; (ii) dietary LNA is sufficiently converted to DHA to increase bioactive lipids derived from DHA in rats [40]; (iii) LNA metabolites can mediate cellular anti-inflammatory effects [41]; (iv) PO-fed rats [40] or cells directly treated with LNA [42] exhibit enhanced β-oxidation, signifying that LNA is a utilizable energy source; (v) long chain ω-3 PUFAs, including EPA and DHA, are formed from LNA via a series of desaturation and elongation reactions [43]; (vi) vegans and vegetarians, who consume negligible DHA, as it primarily originates from plankton-fed fish and marine animals, depend on LNA to provide adequate DHA to their brains [44,45,46]; and finally, (vii) the liver maintains the desaturase and elongase enzymes at their highest concentrations as compared to those in the brain [47,48]. DHA synthesis in the liver occurs at >30-fold higher rates than that in the brain [49]. More interestingly, though the brain is capable of synthesizing DHA [49,50], brain DHA synthesis is ~100-fold lower than brain DHA uptake and consumption rates, indicating that brain DHA synthesis does not contribute significantly to brain DHA homeostasis [51]. This suggests an extra-CNS source of DHA for the brain DHA. However, the proportion of LNA used for the purposes mentioned above must be comprehensively evaluated. If the brain has a limited capacity to convert LNA to DHA (<1%), other tissues including the liver must have a higher conversion capacity, otherwise, the levels of DHA in a vegan’s brain would not increase. Thus, it is conceivable that PO-LNAs may have increased the levels of other lipid mediators, or DHA in the brains of our subjects. However, the mechanism must be known. Unfortunately, we could not directly measure brain levels of EPA or DHA in our current study.

Notably, body weight, BMI, percent body fat, lipid profile (total cholesterol, triglyceride, LDL-cholesterol, HDL-cholesterol), or markers of hepatorenal function (GPT, GOT, γ-GT, ALB) did not differ between PO and placebo groups. Systolic blood pressure decreased by 4.2 mmHg, while diastolic pressure decreased by ~2.5 mmHg in the PO group; however, this was not a significant change. These results suggest that long-term PO administration may have no serious side effects in healthy individuals. PO-administration, however, significantly affected serum levels of NE and serotonin. The changes were accompanied by changes in SDS and apathy scores.

SDS score reflect degree of depression, while apathy scores suggest motivational engagement. However, their symptoms usually overlap. The causal relationship between brain lipids, catecholamines, and depression remain unknown. The serotonin theory of depression suggests that reduced levels of serotonin and/or impaired serotonin receptor/transporter function are core pathogenic factors in depression [52,53]. Consequently, selective serotonin reuptake inhibitors (SSRIs), which usually increase serotonin levels in the synapse, confer anti-depressant activity. Nevertheless, 50% of depressive patients do not response to SSRI therapies, and evidence suggests that adequate serotonin levels are only beneficially therapeutic in moderate to severely depressed patients [52,54,55,56]. Low ω-3 PUFA intake is linked to depression [55], while ω-3 PUFA supplementation enhanced serotonin levels in mice [57] and rats [58]. Previous meta-analytic studies also reported that ω-3 PUFA-intake generally ameliorates depression [59,60]. In contrast, DHA-deficient (25-70% below normal) brains also had elevated levels of serotonin and its receptors [61], suggesting that the effects of ω-3 PUFAs on serotonin are still inconclusive. In this study, reduced SDS score after 12 months of intervention was associated with reduced serum serotonin levels (Figure 3), but serum serotonin levels were not correlated with those of SDS scores (data not shown), suggesting that serum serotonin levels of participants in this study may not be linked to depression.

Additionally, the correlation between ω-3 PUFA and NE levels is yet to be elucidated. Both decreases [62] and lack of change [55,61] in brain NE levels have been observed in the ω-3 PUFA-deficient animal brain. SH-SY5Y neuroblastoma cells pretreated with DHA increased their release of [^3^H]-NE [63]. Hypothetical mechanisms suggest that ω-3 PUFAs induced increases in neuronal membrane fluidity, release of catecholamines, and enhanced membrane-bound enzyme, receptor, and other signal transducing molecule function [64,65]. We have also previously reported that DHA increases the membrane fluidity of synaptosomal plasma membranes [2]. However, the effect of fluidity on catecholamine release and the subsequent action on the human brain and depression seem very complex. In contrast with the effects of ω-3 PUFAs on animal models of depression and/or ω-3 PUFA-deficient animals, there was no prominent increase in serum catecholamine levels in our subjects after 12 months of PO administration. Instead, serum levels of both NE and serotonin significantly decreased in both placebo and PO groups (Figure 3). There are very few studies in this area with which to directly compare our data. We speculate, however, that the changes in catecholamine concentrations brought about an adequate balance between serotonergic and noradrenergic neuronal activity and a neuro-adaptive process. This process ultimately led to a better mental condition in our study subjects, otherwise SDS and apathy scores could not have improved in our PO-intake subjects. Furthermore, PO might have improved the dysregulation of the hypothalamic-pituitary-adrenal axis, which otherwise could have deteriorated the depression and anxiety status of our subjects. Reductions in ω-3 PUFAs, particularly DHA, in the brain have been shown to increase levels of anxiety in mice [66] and rats [67], while dietary ω-3 PUFAs supplementation ameliorated anxiety in rats [68]. Moreover, by decreasing neuroinflammation [9,28,69,70,71], which is often attributed to depression in humans [72], ω-3 PUFAs may have contributed to maintaining healthy mental condition in our subjects.

We need to mention herein the facts that there are some limitations which deserve additional discussion. Firstly, since the participants in this study were healthy individuals without mental disturbance, their mental health parameters were mostly normal and there were no significant differences between PO group and placebo group. Δ-SDS and Δ-Apathy scores were defined as the difference between the baseline and 3 or 12 months in order to achieve statistical differences, since there were no significant differences in both SDS and Apathy scores between the placebo and PO groups. In the future, we should investigate the effects of PO ingestion on individuals with mental disorders such as depression. Secondly, we did not control for menstrual cycle or consider menopause for female subjects, and future research will need to take these into account given the influence of menstrual cycle or menopause on psychological health [73,74,75].

## 5. Conclusions

In conclusion, the current study suggests that PO administration increases blood LNA and EPA levels in the adult subjects, leading to mitigation of mental health issues in adults without having any noticeable clinical side effects. These effects may be a result of the adequate balance between serotonergic and adrenergic neuronal activity. Further investigation is necessary to comprehensively conclude the mechanism responsible for the beneficial effects of PO on mental condition.

## Figures and Tables

**Table 1:** Basic fatty acid composition of placebo and *perilla* oil.

Fatty Acids (g/100 mL)	Placebo	*Perilla* Oil
PLA (C16:0)	9.45 ± 0.05	6.55 ± 0.05
PAL (C16:1, ω-9)	0.20 ± 0.0	0.20 ± 0.00
STA (C18:0)	2.95 ± 0.07	1.60 ± 0.10
OLA (C16:0, ω-9)	39.6 ± 0.07	27.15 ± 0.25
LLA (C18:2, ω-6)	42.0 ± 0.05	14.0 ± 0.75
LNA (C18:3, ω-3)	5.45 ± 0.05	50.4 ± 0.55

PLA: palmitic acid; PAL: palmitoleic acid; STA: stearic acid; OLA: oleic acid; LLA: linoleic acid; LNA: α-linolenic acid. Data are mean ± SE, for triplicate determinations.

**Table 2:** Participant’s base line parameters.

	Placebo (n = 37)				*Perilla* Oil (n = 38)				
	Mean	Median	Min	Max	Mean	Median	Min	Max	*p* Value
Sex (male/female)	16/21				16/22				
Age (years)	47.8	48.0	30	65	48.9	49.0	31	78	0.963
Height (cm)	161.3	162.5	146.6	175.5	161.0	159.6	137.0	174.3	0.727
Body Weight (kg)	61.7	63.3	42.3	91.2	61.3	59.3	40.2	96.0	0.875
BMI (kg/m^2^)	23.6	23.1	16.1	33.4	23.5	23.1	16.2	33.4	0.940
BC (cm)	82.1	83.5	63.0	104.0	83.7	83.3	60.0	108.0	0.526
Body fat (%)	28.4	26.9	11.0	48.2	29.8	30.7	11.4	46.4	0.347
SW (kg)	57.3	57.9	47.3	67.8	57.2	56.1	41.3	66.8	0.754
*Blood Pressure*									
SBP (mmHg)	123.8	127.0	90.0	159.0	124.2	125.0	98.0	160.0	0.790
DBP (mmHg)	76.2	78.0	55.0	98.0	76.1	77.5	58.0	94.0	0.583
*Emotional index*									
SDS score	35.1	34.0	23.0	56.0	36.4	36.5	22.0	54.0	0.373
Apathy score	12.4	12.0	0.0	25.0	14.6	15.0	3.0	26.0	0.205
*Blood biochemistry*									
GOT (U/L)	21.8	21.0	14.0	69.0	22.0	19.0	15.0	48.0	0.821
GPT (U/L)	21.9	19.5	10.0	101.0	22.3	16.0	9.0	83.0	0.820
γ-GT (IU/L)	33.0	25.0	11.0	331.0	39.7	25.0	13.0	189.0	0.808
ALB (g/dL)	4.6	4.6	4.0	5.3	4.6	4.5	4.2	5.3	0.995
TC (mg/dL)	210.7	210.5	90.0	397.0	217.9	216.0	163.0	285.0	0.700
TG (mg/dL)	96.4	85.0	35.0	277.0	101.1	88.0	38	268.0	0.312
BUN (mg/dL)	12.7	12.8	6.8	19.7	12.8	12.7	7.0	24.9	0.740
CRE (mg/dL)	0.8	0.7	0.5	1.1	0.7	0.7	0.6	1.3	0.905
BS (mg/dL)	94.5	94.5	76.0	145.0	95.7	92.0	79.0	161.0	0.667
HDL-C (mg/dL)	65.7	64.0	27.2	108.4	68.4	63.2	44.4	112.8	0.261
LDL-C (mg/dL)	130.4	127.9	19.9	213.0	134.2	130.0	84.0	194.0	0.305
HbA1c (NGSP) (%)	5.7	5.7	5.0	9.1	5.8	5.7	5.1	8.0	0.612
NE (pg/100 μL)	60.9	57.0	24.6	120.0	63.2	55.2	30.8	139.0	0.713
Epi (pg/100 μL)	4.7	3.7	1.5	11.8	5.4	4.3	0.8	20.2	0.596
5-HT (pg/100 μL)	28,839	24,559	8355	75,752	29,652	25,242	10,524	99,019	0.949

BC: belly circumference; BMI: body mass index; SW: standard weight; SBP: systolic blood pressure; DBP: diastolic blood pressure; SDS: self-rating depression scale; GOT: glutamate-oxaloacetate transaminase; GPT: glutamate-pyruvate transaminase; γ-GT: gamma-glutamyl transpeptidase; ALB: albumin; TC: total cholesterol; TG: triglyceride; BUN: blood urea nitrogen; CRE: creatinine; BS: blood sugar; HDL-C: high-density lipoprotein cholesterol; LDL-C: low-density lipoprotein cholesterol; HbA1c: glycated hemoglobin; NE: norepinephrine; Epi: epinephrine; 5-HT: 5-hydroxytryptamine (serotonin). Min: minimum value; Max: maximum value.

**Table 3:** Participant’s parameters after 12 months intervention.

	Placebo (n = 34)				*Perilla* Oil (n = 31)				
	Mean	Median	Min	Max	Mean	Median	Min	Max	*p* Value
Sex (male/female)	14/20				12/19				
Age (years)	49.0	49.2	31	66	50.6	50.0	32	79	0.534
Height (cm)	161.8	162.9	146.7	175.5	160.0	160.0	136.9	174.5	0.308
Body Weight (kg)	62.5	62.1	43.2	93.4	61.1	59.1	40.9	96.5	0.657
BMI (kg/m^2^)	23.8	22.9	17.1	34.1	23.8	23.8	17.3	32.8	0.993
BC (cm)	83.6	83.9	66.0	102.0	84.5	83.6	66.3	109.7	0.741
Body fat (%)	28.4	28.5	17.1	48.1	29.3	30.7	9.5	42.9	0.659
SW (kg)	57.8	58.5	47.2	67.5	56.6	56.0	41.3	66.8	0.334
*Blood Pressure*									
SBP (mmHg)	121.5	126.0	93.0	146.0	120.0	123.0	92.0	151.0	0.673
DBP (mmHg)	74.6	75.5	54.0	90.0	73.7	76.0	54.0	88.0	0.729
*Emotional index*									
SDS score	37.9	36.0	29.0	55.0	37.4	36.0	20.0	61.0	0.805
Apathy score	13.9	13.5	0.0	26.0	12.8	16.0	0.0	21.0	0.496
*Blood biochemistry*									
GOT (U/L)	22.3	21.0	14.0	55.0	23.5	21.0	14.0	52.0	0.529
GPT (U/L)	20.7	17.5	10.0	62.0	20.0	16.0	7.0	66.0	0.814
γ-GT (IU/L)	32.8	23.0	8.0	250.0	40.6	23.0	13.0	314.0	0.531
ALB (g/dL)	4.5	4.6	3.2	5.3	4.6	4.6	4.2	5.0	0.746
TC (mg/dL)	204.0	201.0	143.0	282.0	209.5	206.0	163.0	305.0	0.496
TG (mg/dL)	117.8	79.5	41.0	713.0	99.4	89.0	38.0	307.0	0.435
BUN (mg/dL)	12.6	13.0	5.5	17.6	13.7	13.4	8.2	26.5	0.195
CRE (mg/dL)	0.7	0.7	0.4	1.1	0.7	0.7	0.5	1.4	0.821
BS (mg/dL)	93.3	93.5	71.0	123.0	93.6	92.0	82.0	119.0	0.900
HDL-C (mg/dL)	63.5	59.2	41.6	110.9	66.4	64.5	34.7	111.2	0.515
LDL-C (mg/dL)	123.2	125.5	73.0	177.0	128.8	127.0	87.0	204.0	0.387
HbA1c (NGSP) (%)	5.6	5.5	4.2	6.6	5.7	5.7	4.9	6.6	0.436
NE (pg/100 μL)	42.4	39.3	12.1	100.1	49.3	47.3	20.1	98.9	0.142
Epi (pg/100 μL)	4.7	4.8	1.3	9.8	5.2	4.1	1.5	18.0	0.494
5-HT (pg/100 μL)	22,363	18,460	4697	58,833	21620	20,071	4066	62,820	0.812

BC: belly circumference; BMI: body mass index; SW: standard weight; SBP: systolic blood pressure; DBP: diastolic blood pressure; SDS: self-rating depression scale; GOT: glutamate-oxaloacetate transaminase; GPT: glutamate-pyruvate transaminase; γ-GT: gamma-glutamyl transpeptidase; ALB: albumin; TC: total cholesterol; TG: triglyceride; BUN: blood urea nitrogen; CRE: creatinine; BS: blood sugar; HDL-C: high-density lipoprotein cholesterol; LDL-C: low-density lipoprotein cholesterol; HbA1c: glycated hemoglobin; NE: norepinephrine; Epi: epinephrine; 5-HT: 5-hydroxytryptamine (serotonin). Min: minimum value; Max: maximum value.

**Table 4:** Fatty acid profile of the erythrocyte plasma membranes at baseline and 12 months of *perilla* oil intervention.

	Baseline		12-Months	
(μg/mg Protein)	Placebo n = 37	*Perilla* Oiln = 38	Placebo n = 34	*Perilla* Oil n = 31
PLA	47.8 ± 0.7	49.0 ± 0.8	33.0 ± 0.5	32.9 ± 0.7
STA	41.2 ± 0.7	41.5 ± 0.7	27.8 ± 0.5	28.0 ± 0.8
OLA	32.0 ± 0.5	32.9 ± 0.6	25.8 ± 0.6	25.6 ± 0.5
LLA	26.4 ± 0.5	26.5 ± 0.7	24.0 ± 0.6	23.4 ± 0.6
LNA	0.39 ± 0.02	0.41 ± 0.03	0.42 ± 0.03	0.67 ± 0.05*
AA	30.3 ± 0.6	30.8 ± 0.5	23.9 ± 0.7	23.2 ± 0.6
EPA	2.8 ± 0.2	2.9 ± 0.2	2.2 ± 0.2	2.4 ± 0.1
DPA	4.9 ± 0.1	4.8 ± 0.1	3.1 ± 0.1	3.2 ± 0.1
C24:0	7.4 ± 0.3	7.6 ± 0.2	9.7 ± 0.2	9.6 ± 0.2
DHA	16.1 ± 0.4	16.3 ± 0.4	15.2 ± 0.6	15.0 ± 0.5
C24:1	7.3 ± 0.2	7.7 ± 0.2	7.4 ± 0.2	7.1 ± 0.2
Total	217.1 ± 3.4	220.9 ± 3.4	173.4 ± 3.1	172.0 ± 3.3

PLA: palmitic acid; STA: stearic acid; OLA: oleic acid; LLA: linoleic acid; LNA: α-linolenic acid; AA: arachidonic acid; EPA: eicosapentaenoic acid; DPA: docosapentaenoic acid; DHA: docosahexaenoic acid; Data are mean ± SE. * *p* < 0.05 vs. placebo.

## Supplementary Materials

The following are available online at https://www.mdpi.com/2304-8158/9/4/530/s1: Figure S1: The differences between male and female in Δ-SDS score from baseline to 12 months in the PO and placebo groups.
