# Supplementary material for: Twelve-Month Studies on Perilla Oil Intake in Japanese Adults—Possible Supplement for Mental Health
Source: Foods. 2020 Apr 22;9(4):530. doi: 10.3390/foods9040530 (PMC7230189; doi:10.3390/foods9040530)
Supplement: Supplementary file 1 [file foods-09-00530-s001.pdf]

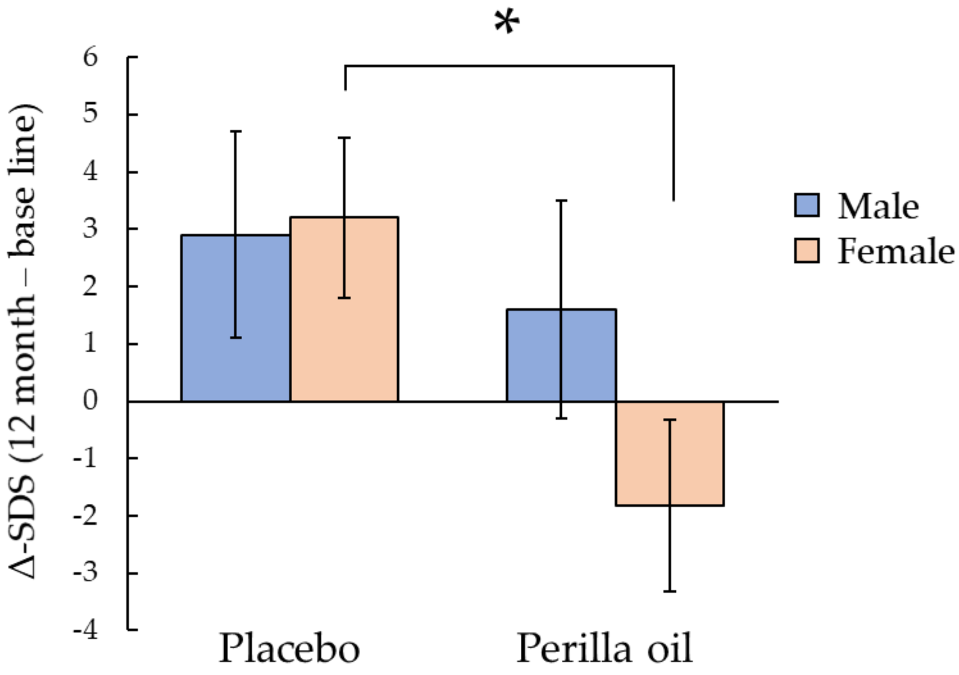

Supplemental Figure S1. The differences between male and female in Δ-SDS score from baseline to 12 months in the PO and placebo groups. Δ-SDS score in female subjects was significantly lower in the PO group than that of placebo group. Results are means ± SE. *$p < 0.05$.
